# Enhanced production of alkane hydroxylase from *Penicillium chrysogenum* SNP5 (MTCC13144) through feed-forward neural network and genetic algorithm

**DOI:** 10.1186/s13568-022-01366-1

**Published:** 2022-03-03

**Authors:** Satyapriy Das, Sangeeta Negi

**Affiliations:** Department of Biotechnology, Motilal Nehru National Institute of Technology Allahabad, Prayagraj, U.P. 211004 India

**Keywords:** Alkane hydroxylase (AlkB), Feed Forward Neural Network (FFNN), Genetic algorithm (GA), *Penicillium chrysogenum* SNP5, Submerged fermentation (SmF)

## Abstract

Alkane hydroxylase (AlkB), a membrane-bound enzyme has high industrial demand; however, its economical production remains challenging due to its intrinsic nature and co-factor dependency. In the current study, various critical process parameters for optimum production of AlkB have been optimized through feed forward neural network (FFNN) and genetic algorithm (GA) models using *Penicillium chrysogenum* SNP5 (MTCC13144). AlkB specific activity under preliminary un-optimized conditions i.e., 1% hexadecane, 7.4 pH, 11 days incubation time, 28 °C incubation temperature and 1 ml of inoculum size was 100 U/mg. ‘One variable at a time’ (OVAT) strategy was used to identify optimum physicochemical parameters and then its output data was fed to develop a model of FFNN with ‘6-12-1’ topology. Outputs of FFNN were further optimized through GA to minimize errors and intensify search level. This has provided superior predictive performances with 0.053 U/mg overall mean absolute percentage error (MAPE), 6.801 U/mg root mean square errors (RMSE), and 0.987 overall correlation coefficient (R). The AlkB specific activity improved by 3.5-fold, i.e., from 100 U/mg under preliminary un-optimized conditions to 351.32 U/mg under optimum physicochemical conditions obtained through FFNN-GA hybrid method, i.e., hexadecane (carbon source): 1.56% v/v, FeSO_4_: 0.63 mM, incubation temperature: 27.40 °C, pH: 7.38, incubation time: 12.35 days and inoculums size: 1.33 ml. The developed process would be a stepping stone to fulfill the high industrial demands of  Alkane hydroxylase.

## Introduction

The membrane-bound alkane hydroxylase (AlkB) is a versatile biocatalyst that introduces molecular oxygen in inert alkanes with regio and stereoselectivity. The AlkB system is comprised of three subunits: AlkB, soluble rubredoxin reductase, and soluble rubredoxin. AlkB incorporates molecular oxygen from O_2_ to the alkane and the remaining oxygen gets reduced to water by electrons released from rubredoxin on the action of rubredoxin reductase (Eidani et al. 2012). Various divergent forms of Alkane hydroxylases ubiquitously found worldwide viz. soluble methane monooxygenase (sMMO) and copper-containing methane monooxygenase (pMMO) are capable of oxidizing hydrocarbons ranging from C_1_ to C_8_ (Van Beilen and Funhoff 2007). While, integral membrane-bound AlkB insert oxygen in C_5_ to C_16_ (Aliakbari et al. 2014), other forms such as cytochrome P450, LadA, or AlmA assimilate oxygen to alkanes larger than C_20_ (Wang and Shao 2012) and sometimes opt for medium-chain alkanes as substrate (Xu et al. 2015).

AlkB has tremendous market demand in synthesizing industrially important molecules such as secondary metabolites, steroids, polyketides, pharmaceutical compounds, cosmetics, fragrance and agrochemical intermediates, etc. (Rojo 2010; Ramu et al. 2012). It has some other promising applications like conversion of petroleum waste into activated intermediates, bioremediation and biotransformation. In general, either whole cell or partially purified AlkB may be used in biotransformation depending upon the requirements. Ramu et al. (2012) explored this enzyme in a whole-cell biotransformation system by recombinant expression in *Escherichia coli* to regioselectively synthesize 2,2-, 3,3- and 4,4-difluorooctan-1-ols, from simple and inexpensive starting materials. However, opting for whole cells for oxidation becomes challenging due to the slow uptake of a lipophilic substrate which results in the production of toxic compounds and a low oxygen transfer rate (Ayala and Toress 2004). Alkane hydroxylases have been well explored in therapeutics also, where it is used to treat inflammation, vascular liver diseases and peroxisome disorders of fatty acid metabolism.

The alkane hydroxylase system is distributed in a wide range of bacterial strains (*Burkholderia, Pseudomonas*, *Acinetobacter, Alcanivorax,* and *Rhodococcus* strains) and a few fungal strains like *Aspergillus* sp. (Nie et al. 2014; Kadri et al. 2018). Its overproduction has been achieved through overexpressing the all gene of both the gram-negative and gram-positive bacteria into another host; however, rubredoxin and rubredoxin reductase were reported as their essential cofactors (Luo et al. 2015). It has been reported by Kadri et al. (2018) and Al-Hawash et al. (2018) that media engineering and selection of appropriate carbon sources improved AlkB specific activity in *Alcanivorax borkumensis* and *Aspergillus *sp. RFC-1. From the results and observations of earlier studies, it was observed that the bottleneck of the process is its intrinsic nature and cofactor dependency. Therefore, to overcome these bottlenecks various interdisciplinary approaches and techniques have to be put together to achieve optimum specific activity of AlkB economically. Being membrane-bound, its yield remains dependent on cell growth and concentration; and further cofactors are essential for its functions, hence, its yield could be enhanced through optimizing the physicochemical critical parameters, which influence cell growth most. The global scientific and industrial world has been witnessing the increasing use of FFNN and GA together for process optimization for enhanced product yield.

An experimental design, i.e., screening and optimization design is considered pivotal to computer-assisted design-guided statistical exercise. The aims of factor screening and optimization can be accomplished by opting for a design-guided experimental strategy using selected experimental designs. Experimental designs are modeled by selecting appropriate mathematical models like linear, quadratic and cubic to generate 2D and 3D-response variables to figure out inter and intra factorial interactions. To search for optimum yield or solution, various numerical and graphical optimization techniques such as FFNN, desirability function and overlay plot are opted, which are located in design and control spaces. Design space is a multidimensional combination of input and response variables to determine the optimal solution with high accuracy and quality.

In bioprocesses optimization (Negi et al. 2020), pattern recognition in spectrum data, functional analysis of genomes and proteomes (Wardah et al. 2019) and their nonlinear functions are designed through FFNN (May et al. 2011). Many studies reported that FFNN has better efficiency, accuracy and yield as compared to the other statistical optimization methods such as RSM (Prakash Maran and Priya 2015). Genetic algorithms (GAs) are randomly determined search methods based on some basic operations like selection, reproduction or crossover, and mutation as natural genetics to find out the best fitness value/outcome (Murthy 2012). GA has also been well explored by many researchers to achieve optimum process parameters for enhancing product yield in various biological systems (Kana et al. 2012). In previous studies, coupled FFNN-GA system has been effectively used for optimizing the production of cellulase (Chang et al. 2011) and glutaminase (Sathish and Prakasham 2010). These studies concluded that FFNN-GA coupled system has better proficiency with minimum errors compared to the other optimization methodologies. Hence FFNN-GA coupled system is emerging as an effective tool in optimization studies (Singh et al. 2017).

In the present study, FFNN-GA coupled system was used to optimize fermentation parameters to achieve maximum specific activity of AlkB from *Penicillium chrysogenum* SNP5. FFNN was used for the training of experimental data and GA was used for the optimization of input variables further with the help of weight and biases generated from the neural network.

## Material and methods

### Microorganism and media chemicals

*Penicillium chrysogenum* SNP5 (MTCC13144) strain was locally isolated from grease contaminated soil of the diesel loco shed and identified by Microbial Type Culture Collection, Chandigarh, India. Its ITS/5.8S rRNA and the β-tubulin gene sequences have been submitted to GenBank and Bankit with accession numbers: OL336466 and OL352703 respectively. Triton X-100, Phenylmethylsulfonyl fluoride (PMSF), Lauryldimethylamine oxide (LDAO), Nicotinamide adenine dinucleotide (NAD) + hydrogen (NADH), other medium components and Czapek-dox medium were procured from Sisco Research Laboratories Pvt. Ltd. (Mumbai, India). Hexadecane, Digitonin and THB (Tetrahydrobiopterin) were procured from TCI Chemicals Pvt. Ltd. (India).

### Production of alkane hydroxylase under submerged fermentation (SmF)

Production of AlkB was performed in a 250 ml Erlenmeyer flask with a working volume of 100 ml. Initially, hexadecane 1% (v/v), 0.5% YEPD, 0.1% glucose and 1 mM TBH were dissolved in 50 ml of Czapek-dox broth (NaNO_3_—2.5 g/l, KH_2_PO_4_—1.0 g/l, MgSO_4_⋅7H_2_O—0.5 g/l, KCl—0.5 g/l, FeSO_4_—0.45 g/l) and final volume of 100 ml was maintained by using distilled water. The flasks were autoclaved at 121 °C, 121psi for 15 min. *Penicillium chrysogenum* SNP5 (MTCC13144) strain was cultivated on potato dextrose agar (PDA) slant and incubated for 6–7 days. The inoculum was prepared with sterile distilled water by maintaining the spore’s concentration of 1.4 × 10^7^ spores/ml. Each flask was inoculated with 1.0 ml of spore suspension and incubated at 28 °C for 11 days, and growth was observed.

### Extraction of AlkB and its activity measurement

After fermentation, the cells were harvested by centrifuging the fermented broth at 7826*g* for 10 min to separate the cells. The cell pellets containing AlkB were washed two times with Tris–HCl buffer (pH 7.4) and lysed to recover the enzyme by using ultrasonicator (Model SKL-500D) Ningbo Haishu Sklon Electronics Instrument Co, Ltd. (Mainland, China) at 70 kHz using 9 s on 9 s off pulsating cycle for 5 min in lysis buffer (150 mM NaCl, 20% (v/v) glycerol, 50 mM Tris HCl, 1 mM digitonin, 2% Triton X-100 and 1 mM PMSF) and then centrifuged at 11,269*g* for 15 min. The clear supernatant was collected, and the remaining cell pellet was resonicated followed by centrifugation, and both the supernatant were pooled to serve as crude AlkB enzyme for further study.

AlkB activity in the crude extract was measured by a continuous method using NADH as cofactor and hexadecane as substrate by a modified protocol of McKenna and Coon (1970). Reaction mixture contained 100 mM Tris HCl buffer (pH—7.4), 0.035% LDAO (1.5 CMC), 20% glycerol, 100 μl crude enzyme, 1 mM hexadecane. A mixture lacking NADH was used as a negative control. The reaction mixture was then incubated at room temperature for 20 min. The reaction was initiated by adding NADH to a final concentration of 50 μM. The rate of NADH consumption was determined by monitoring the change in absorbance at 340 nm at room temperature for 10 min. One unit is defined as the amount of enzyme required for the consumption of 1 μM of NADH (ε340 = 6220 M^−1^ cm^−1^) per min.

### Optimization of process parameters by OVAT method

SmF optimization experiments were planned according to OVAT method with six selected process parameters as mentioned in Table 1 i.e., hexadecane concentration (as carbon source), incubation temperature, pH of the media, incubation time, inoculum size and metal ion concentration (FeSO_4_). Tested ranges of variables were hexadecane (0.25–4% v/v), temperature (20–38 °C), pH (4–9), incubation time (5–14 days), inoculum size (0.25–3.0 ml) and FeSO_4_ (0.05–0.8 mM).

**Table 1:** Selected parameters for optimization through ‘OVAT’ method for AlkB by *Penicillium chrysogenum* SNP5 under SmF

Variables code	Variables name	Lower bound	Upper bound
V_1_	Hexadecane %	0.25	4
V_2_	Temperature (°C)	20	38
V_3_	pH	4	9
V_4_	Incubation time (Days)	5	14
V_5_	Inoculum size (ml)	0.25	3
V_6_	FeSO_4_ conc. (mM)	0.05	0.8

### Modeling of optimization process by FFNN

The feed-forward neural network along with the back-propagation learning algorithm has been employed in this study to optimize nonlinear data obtained from the ‘one variable at a time’ method and to reduce the experimental error. The network consists of input, hidden and output layers along with additional nodes termed as the bias (bias^I^ and bias^H^). The connection between each layer has been denoted as weight (weight^H^ and weight^O^). Tan sigmoid functions have been used for optimum output. The network outcomes such as weights and biases have been expressed with the following equation (Norgaard 2000; Izadifar 2005).

In the present study, six process parameters were selected, i.e., hexadecane (carbon source) concentration, temperature, pH, incubation time, inoculum size and metal ion concentration (i.e., FeSO_4_) to enhance the AlkB productivity. Process parameters were initially optimized with the ‘OVAT’ method by considering the upper and lower limits of each parameter (Table 1). Total 47 experimental sets were performed using the OVAT method, and later it was extended upto 103 sets using the regression equation. Out of 103 sets, 73 (~ 70%) were selected for training, 15 (~ 15%) were used for validation, and the rest of the 15% data were used for testing in FFNN modeling (Table 2). The neural network was trained by using MATLAB R2020a (The MathWorks, Inc., Natick, MA, USA). Levenberg–Marquardt (trainlm) algorithm was used and numbers of hidden neurons were increased one by one to obtain the best correlation. The best training run was determined by the coefficient R for training, validation and test, which describes the extent of back-propagation in the modeled network. The mean absolute percentage error (MAPE) and root mean square error (RMSE) were calculated using the experimental output and predicted output as described previously (Zhang and Fang 2006).

**Table 2:** Experimental ‘OVAT’ data and FFNN predicted data for AlkB specific activity

S. no.	V1	V2	V3	V4	V5	V6	Experimental AlkB specific activity (U/mg)	Predicted AlkB specific activity (U/mg)	Error
1	0.25	28	7	9	1	0.1	64.8	57.17	7.62
2	0.5	28	7	9	1	0.1	74.53	69.17	5.35
3	0.75	28	7	9	1	0.1	88.38	84.82	3.55
4	1	28	7	9	1	0.1	97.41	100.50	− 3.09
5	1.25	28	7	9	1	0.1	100.45	112.56	− 12.1
6	1.5	28	7	9	1	0.1	117.93	119.80	− 1.87
7	1.75	28	7	9	1	0.1	114.43	123.10	− 8.67
8	2	28	7	9	1	0.1	112.64	123.85	− 11.2
9	2.25	28	7	9	1	0.1	112.79	123.14	− 10.3
10	2.5	28	7	9	1	0.1	112.51	121.64	− 9.13
11	2.75	28	7	9	1	0.1	112.12	119.70	− 7.58
12	3	28	7	9	1	0.1	111.77	117.50	− 5.73
13	3.25	28	7	9	1	0.1	111.56	115.11	− 3.55
14	3.5	28	7	9	1	0.1	111.66	112.55	− 0.89
15	3.75	28	7	9	1	0.1	110.97	109.79	1.17
16	4	28	7	9	1	0.1	110.39	106.78	3.60
17	1.5	20	7	9	1	0.1	75.52	78.51	− 2.99
18	1.5	21	7	9	1	0.1	80.33	79.05	1.27
19	1.5	22	7	9	1	0.1	84.23	80.77	3.45
20	1.5	23	7	9	1	0.1	87.65	84.76	2.88
21	1.5	24	7	9	1	0.1	93.66	93.06	0.59
22	1.5	25	7	9	1	0.1	100.71	108.41	− 7.70
23	1.5	26	7	9	1	0.1	120.44	130.55	− 10.11
24	1.5	27	7	9	1	0.1	142.25	140.41	1.83
25	1.5	28	7	9	1	0.1	140.44	119.80	20.63
26	1.5	29	7	9	1	0.1	138.33	122.19	16.13
27	1.5	30	7	9	1	0.1	137.13	133.15	3.976
28	1.5	31	7	9	1	0.1	136.77	135.37	1.39
29	1.5	32	7	9	1	0.1	135.56	129.16	6.39
30	1.5	33	7	9	1	0.1	123.44	117.46	5.97
31	1.5	34	7	9	1	0.1	96.46	102.78	− 6.32
32	1.5	35	7	9	1	0.1	93.22	86.96	6.25
33	1.5	36	7	9	1	0.1	60.6	71.36	− 10.76
34	1.5	37	7	9	1	0.1	42.55	56.90	− 14.35
35	1.5	38	7	9	1	0.1	42.02	44.13	− 2.11
36	1.5	27	4	9	1	0.1	39.46	34.51	4.94
37	1.5	27	4.25	9	1	0.1	40.13	40.10	0.021
38	1.5	27	4.5	9	1	0.1	41.9	45.22	− 3.32
39	1.5	27	4.75	9	1	0.1	43.55	49.65	− 6.10
40	1.5	27	5	9	1	0.1	53.46	53.34	0.11
41	1.5	27	5.25	9	1	0.1	56.67	56.43	0.23
42	1.5	27	5.5	9	1	0.1	66.75	59.41	7.33
43	1.5	27	5.75	9	1	0.1	67.89	63.67	4.21
44	1.5	27	6	9	1	0.1	73.71	72.40	1.30
45	1.5	27	6.25	9	1	0.1	87.43	90.08	− 2.65
46	1.5	27	6.5	9	1	0.1	110.85	114.85	− 4.00
47	1.5	27	6.75	9	1	0.1	132.23	134.05	− 1.82
48	1.5	27	7	9	1	0.1	148.27	140.41	7.85
49	1.5	27	7.25	9	1	0.1	137.76	137.13	0.62
50	1.5	27	7.5	9	1	0.1	135.68	128.47	7.20
51	1.5	27	7.75	9	1	0.1	121.3	116.76	4.53
52	1.5	27	8	9	1	0.1	104.81	103.18	1.62
53	1.5	27	8.25	9	1	0.1	79.55	88.59	− 9.04
54	1.5	27	8.5	9	1	0.1	69.19	73.74	− 4.55
55	1.5	27	8.75	9	1	0.1	43.66	59.37	− 15.71
56	1.5	27	9	9	1	0.1	39.44	46.18	− 6.74
57	1.5	27	7	5	1	0.1	80.56	89.64	− 9.08
58	1.5	27	7	5.5	1	0.1	96.34	94.52	1.81
59	1.5	27	7	6	1	0.1	99.39	99.97	− 0.58
60	1.5	27	7	6.5	1	0.1	100.34	105.99	− 5.65
61	1.5	27	7	7	1	0.1	115.79	112.58	3.25
62	1.5	27	7	7.5	1	0.1	120.93	119.47	1.45
63	1.5	27	7	8	1	0.1	139.98	126.62	13.35
64	1.5	27	7	8.5	1	0.1	141.32	133.71	7.605
65	1.5	27	7	9	1	0.1	143.06	140.41	2.64
66	1.5	27	7	9.5	1	0.1	144.12	146.32	− 2.20
67	1.5	27	7	10	1	0.1	144.5	151.07	− 6.57
68	1.5	27	7	10.5	1	0.1	142.11	154.29	− 12.19
69	1.5	27	7	11	1	0.1	148.6	155.75	− 7.15
70	1.5	27	7	11.5	1	0.1	148.93	155.37	− 6.44
71	1.5	27	7	12	1	0.1	155.5	153.30	2.19
72	1.5	27	7	12.5	1	0.1	149.45	149.97	− 0.52
73	1.5	27	7	13	1	0.1	138.5	146.00	− 7.50
74	1.5	27	7	13.5	1	0.1	136.89	142.21	− 5.32
75	1.5	27	7	14	1	0.1	136.96	139.58	− 2.62
76	1.5	27	7	12	0.25	0.1	133.65	130.72	2.92
77	1.5	27	7	12	0.5	0.1	146.44	139.22	7.21
78	1.5	27	7	12	0.75	0.1	147.66	147.40	0.253
79	1.5	27	7	12	1	0.1	150.2	153.30	− 3.10
80	1.5	27	7	12	1.25	0.1	155.65	155.77	− 0.126
81	1.5	27	7	12	1.5	0.1	162.25	155.13	7.119
82	1.5	27	7	12	1.75	0.1	161.44	153.36	8.07
83	1.5	27	7	12	2	0.1	160.94	153.25	7.68
84	1.5	27	7	12	2.25	0.1	160.11	154.93	5.17
85	1.5	27	7	12	2.5	0.1	160.33	154.35	5.97
86	1.5	27	7	12	2.75	0.1	158.45	149.29	9.15
87	1.5	27	7	12	3	0.1	150.77	141.48	9.28
88	1.5	27	7	12	1.5	0.05	147.44	148.59	− 1.15
89	1.5	27	7	12	1.5	0.15	158.33	160.52	− 2.19
90	1.5	27	7	12	1.5	0.1	164.06	155.13	8.92
91	1.5	27	7	12	1.5	0.25	164.33	172.13	− 7.80
92	1.5	27	7	12	1.5	0.2	165.1	165.91	− 0.81
93	1.5	27	7	12	1.5	0.35	169.43	185.65	− 16.2
94	1.5	27	7	12	1.5	0.3	185.38	179.12	6.25
95	1.5	27	7	12	1.5	0.45	184.32	191.79	− 7.47
96	1.5	27	7	12	1.5	0.4	198.94	190.09	8.84
97	1.5	27	7	12	1.5	0.5	198	191.66	6.33
98	1.5	27	7	12	1.5	0.55	197.37	191.21	6.15
99	1.5	27	7	12	1.5	0.6	192.69	191.37	1.31
100	1.5	27	7	12	1.5	0.65	193.44	192.27	1.160
101	1.5	27	7	12	1.5	0.7	195.56	193.77	1.78
102	1.5	27	7	12	1.5	0.75	194.33	195.68	− 1.35
103	1.5	27	7	12	1.5	0.8	197.06	197.92	− 0.86

### GA optimization

Optimization with a GA was carried out using FFNN outputs (i.e., weights and biases) by assigning fitness functions to each population. The global optimum was localized on objective function using genetic algorithm outputs. Our main objective was to find optimum input variables for the highest specific activity of AlkB by fixing the lower and upper bound of input variables (Table 1). For optimization of neural network output, the GA toolbox of MATLAB R2020a (The MathWorks, Inc., Natick, MA, USA) was used to achieve optimum conditions in the given range of input variables. GA optimization parameters, i.e., population size as default (i.e., 50 for five or fewer otherwise 200), crossover probability (i.e., 0.8), mutation probability (i.e., 0.01) and the maximum number of generations (i.e., 500) were considered based on literature (Prakasham et al. 2011).

## Results

Our previous studies revealed that *Penicillium chrysogenum* SNP5 (MTCC13144) has great potential for conversion of hydrocarbons of complex grease waste into fatty acids (Kumar et al. 2012; Kumari et al. 2017). Therefore, in the current study, *Penicillium chrysogenum* SNP5 has been explored for the production of AlkB, which is a key player in the uptake of hydrocarbons as a carbon source. AlkB specific activity is growth associated, therefore, depends on various fermentation parameters. In this study, an initial experimental setup was done by taking 1% (v/v) hexadecane, 0.5% (w/v) YEPD, 0.1% (w/v) glucose as carbon source, and 1 mM TBH as a modulator for inducing higher production of AlkB under physicochemical conditions of pH (7.4), incubation time (11 days), incubation temperature (28 °C) and inoculum size (1 ml). The maximum AlkB specific activity found with this setup was 100 U/mg. Further, to find optimum conditions for improved AlkB specific activity, these parameters were varied between the assigned lower and upper bounds (Table 1), and the experimental layout was prepared by the ‘OVAT’ method (Irfan et al. 2014) where one factor was varied at a time keeping others constant (Table 2). A total of 103 experimental sets were obtained and the data were analyzed using an FFNN, where predicted values were compared with experimental outputs. It was observed that the AlkB specific activity altered with variation in different fermentation conditions (Table 2).

**Fig. 1:**
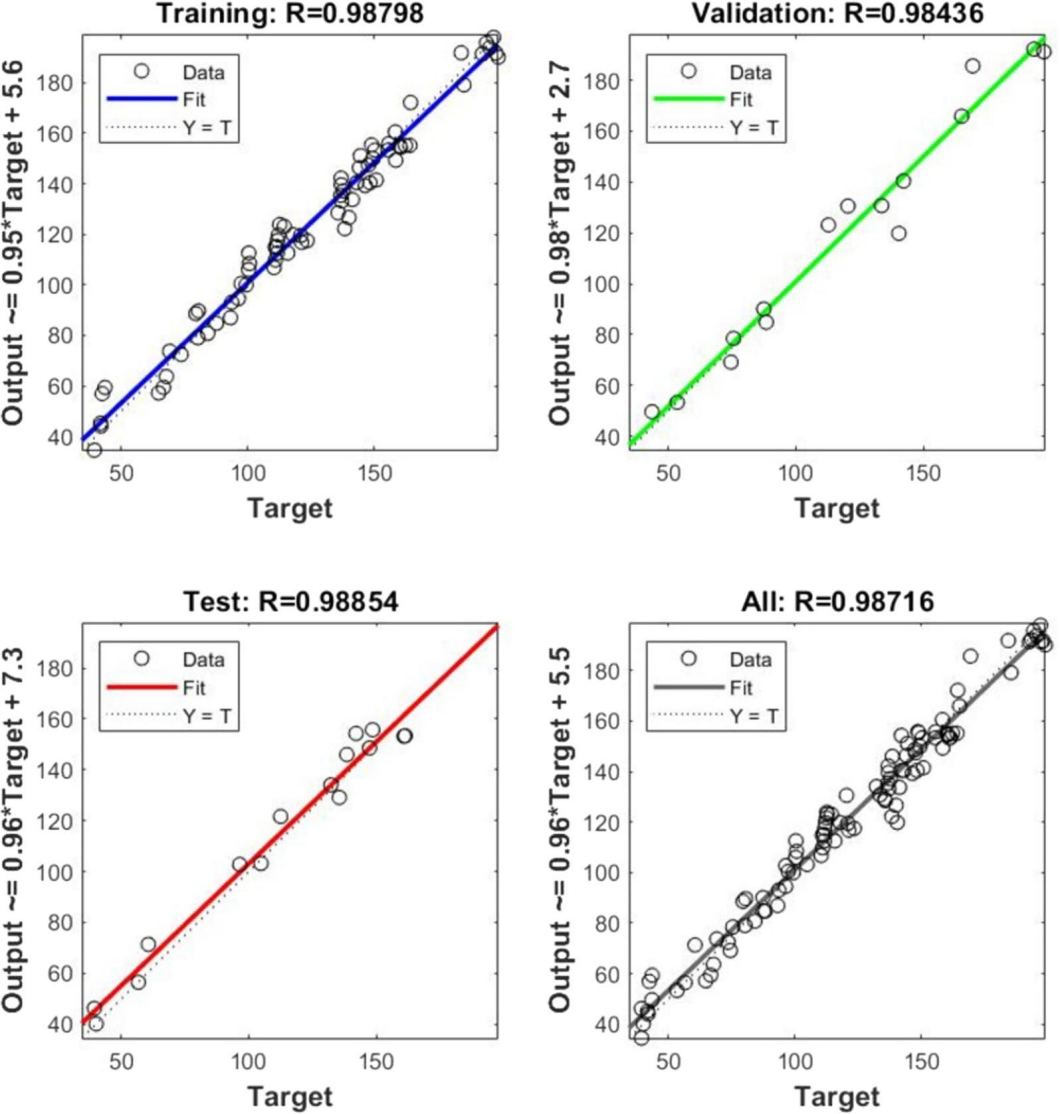
Correlation chart of feed forward neural network predicted and experimental AlkB specific activity

**Fig. 2:**
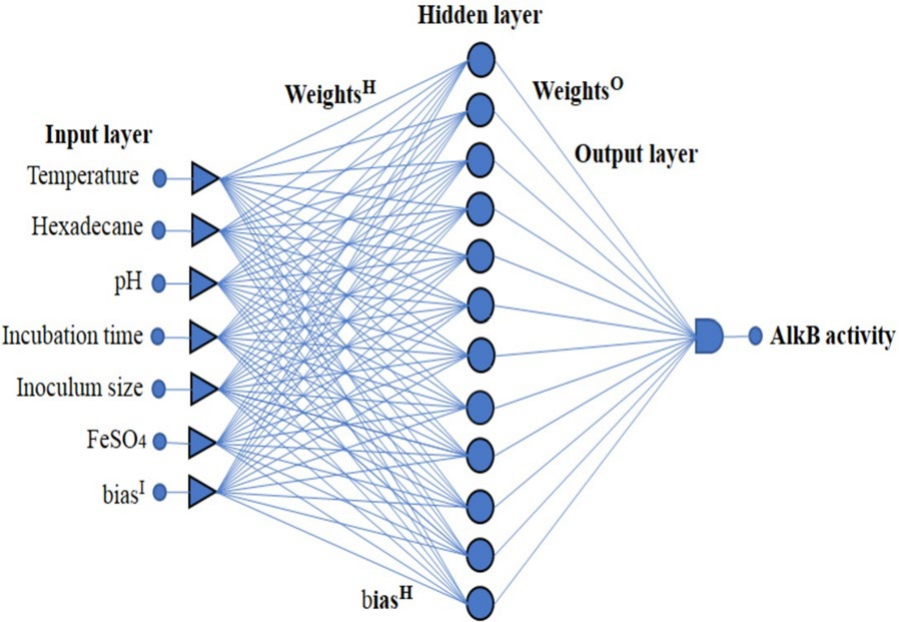
Feed forward neural network architecture (i.e., 6-12-1 topology)

Further, FFNN was trained one by one with Levenberg–Marquardt (Rumelhart et al. 1986), Bayesian (MacKay 1992), Conjugate (Powell 1977), and scaled conjugate gradient (Moller 1993) methods. Out of all these methods, Levenberg–Marquardt back-propagation with trainlm algorithm showed a better R value (i.e., for Training: 0.987, validation: 0.984, test: 0.988, and overall: 0.987) shown in (Fig. 1) along with experimental outputs (41.9 to 198.94 U/mg) and simulated output (39.14 to 200.03 U/mg). The mean absolute percentage error (MAPE) and root mean square error (RMSE) observed were 0.053 and 6.801 U/mg. The final weights and biases values were optimized by minimizing the network error (Table 3), and the optimum result was found in a ‘6-12-1’ FFNN network topology for this study (Fig. 2).

**Table 3:** The weight and bias values obtained from the FFNN at optimum conditions

Weight between input and hidden nodes (IW)						Bias I	Weight between hidden and output nodes (LW)	Bias H
V_1_	V_2_	V_3_	V_4_	V_5_	V_6_			
1.01	1.275	0.446	0.372	0.756	0.814	− 2.482	1.219	0.0281
− 0.915	− 0.41	2.135	− 0.075	− 0.329	− 2.589	− 0.595	0.207	
− 1.579	0.538	0.895	1.921	− 1.05	− 1.047	2.614	0.945	
1.18	− 0.74	− 0.813	− 1.187	0.707	1.109	− 1.57	− 0.123	
0.33	− 0.499	0.329	0.110	0.407	0.538	− 0.121	− 0.264	
− 2.62	1.682	− 5.4	0.632	0.96	− 0.507	− 0.584	− 0.669	
0.032	− 0.016	2.562	3.110	2.761	2.33	− 1.32	0.332	
− 0.202	3.281	0.284	0.887	0.009	0.626	1.136	1.533	
− 0.097	− 1.15	− 1.61	− 1.448	− 0.303	− 1.302	− 0.282	0.766	
0.295	− 9.422	− 1.546	− 0.158	1.4	1.23	0.532	0.766	
− 0.825	1.111	− 0.611	− 0.107	0.49	1.122	− 2.267	0.237	
0.928	1.386	0.295	0.377	− 0.702	− 0.658	2.606	− 0.794

**Fig. 3:**
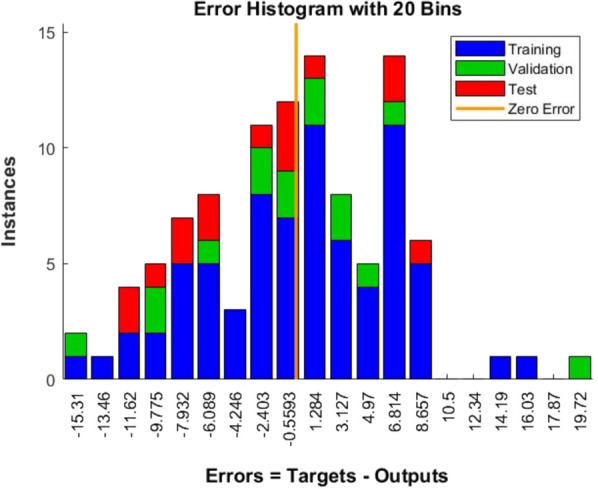
Performance plot obtained after feed-forward neural network training

**Fig. 4:**
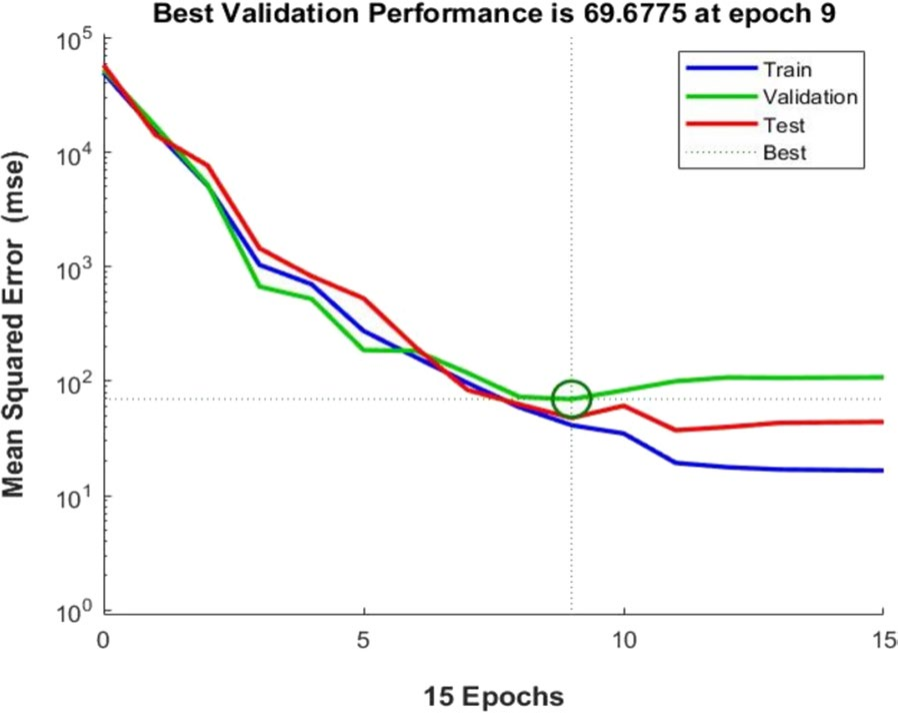
Error histogram obtained after feed forward neural network training

The network performance by mean square error (MSE) (Fig. 3) and network error by error histogram (Fig. 4) were also analyzed. The network performance plot shows that the mean square error for training, validation and test converged after the 9th epoch (Fig. 3). The error histogram showed that most of the training errors occurred between − 9.77 and 6.8 values (Fig. 4). The overall outcome of the neural network showed the goodness of the ‘6-12-1’ neural topology and excellent correlation to train the input parameter data for the production of AlkB.

The outputs obtained from a neural network were optimized to get the best optimum input parameters for maximum AlkB specific activity using a genetic algorithm because existing algorithms possess only local optimization solutions for a nonlinear function, whereas, a genetic algorithm exhibits a global solution. After large numbers of genetic algorithms trials, the five best input conditions were selected (Table 4), which could depict the fittest possible input conditions. All these conditions were employed for further verification by setting up experiments, followed by the comparison of experimental AlkB specific activity with genetic algorithm outputs. An increase in AlkB specific activity was observed by 77.4% (198.94 to 351.32 U/mg) when FFNN outputs were optimized with GA.

**Table 4:** The fittest optimum process parameters optimized with a genetic algorithm and verified AlkB specific activity

No. of GA runs	V_1_	V_2_	V_3_	V_4_	V_5_	V_6_	GA-optimized AlkB specific activity (U/mg)	Experimental AlkB specific activity (U/mg)	No. of iterations
1st	1.92	27.41	7.38	10.27	1.250	0.73	338.61	314.68	128
2nd	1.56	27.40	7.38	12.35	1.331	0.63	355.86	351.32	161
3rd	1.36	27.34	7.41	12.31	1.327	0.70	365.46	336.54	126
4th	1.32	27.42	7.34	11.56	1.325	0.64	363.39	339.67	153
5th	1.46	27.46	7.42	12.32	1.192	0.70	345.12	331.45	142

**Fig. 5:**
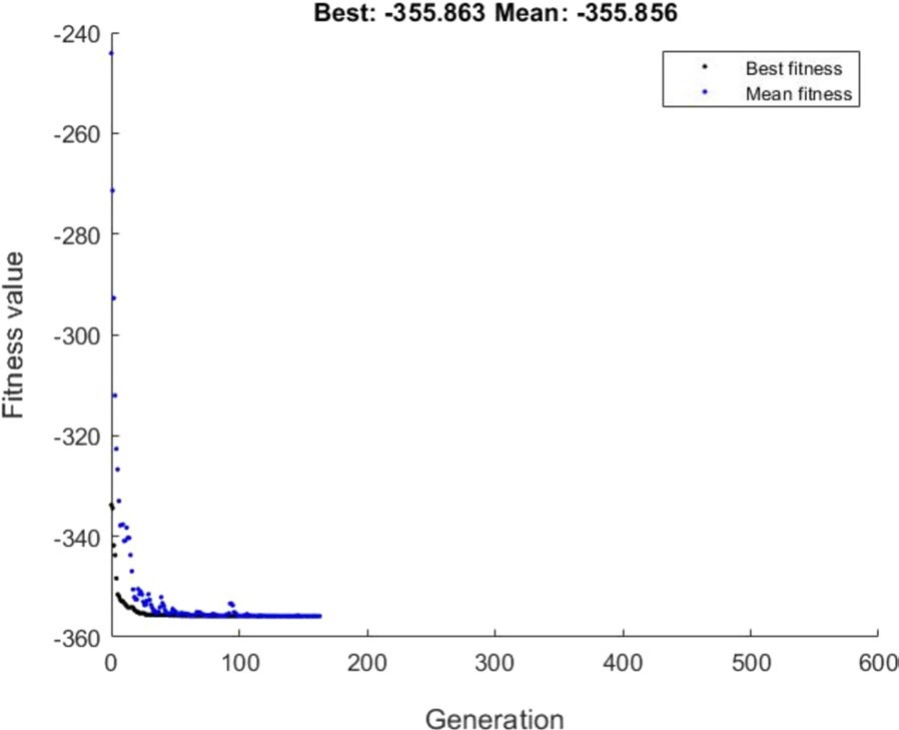
Parameters obtained from GA optimization, shows the fittest value of AlkB specific activity at 161th iterations

Figure 5 depicts the optimum output of GA optimization along with the contribution of each variable where fitness values were expressed in terms of the mean value. The best fitness was obtained at the 161th generation at which fitness value and mean value were found aligned at a constant rate. Similar results have been reported by Subba Rao et al. (2008) for optimization of protease yield and Pappu and Gummadi (2017) for xylitol production.

**Fig. 6:**
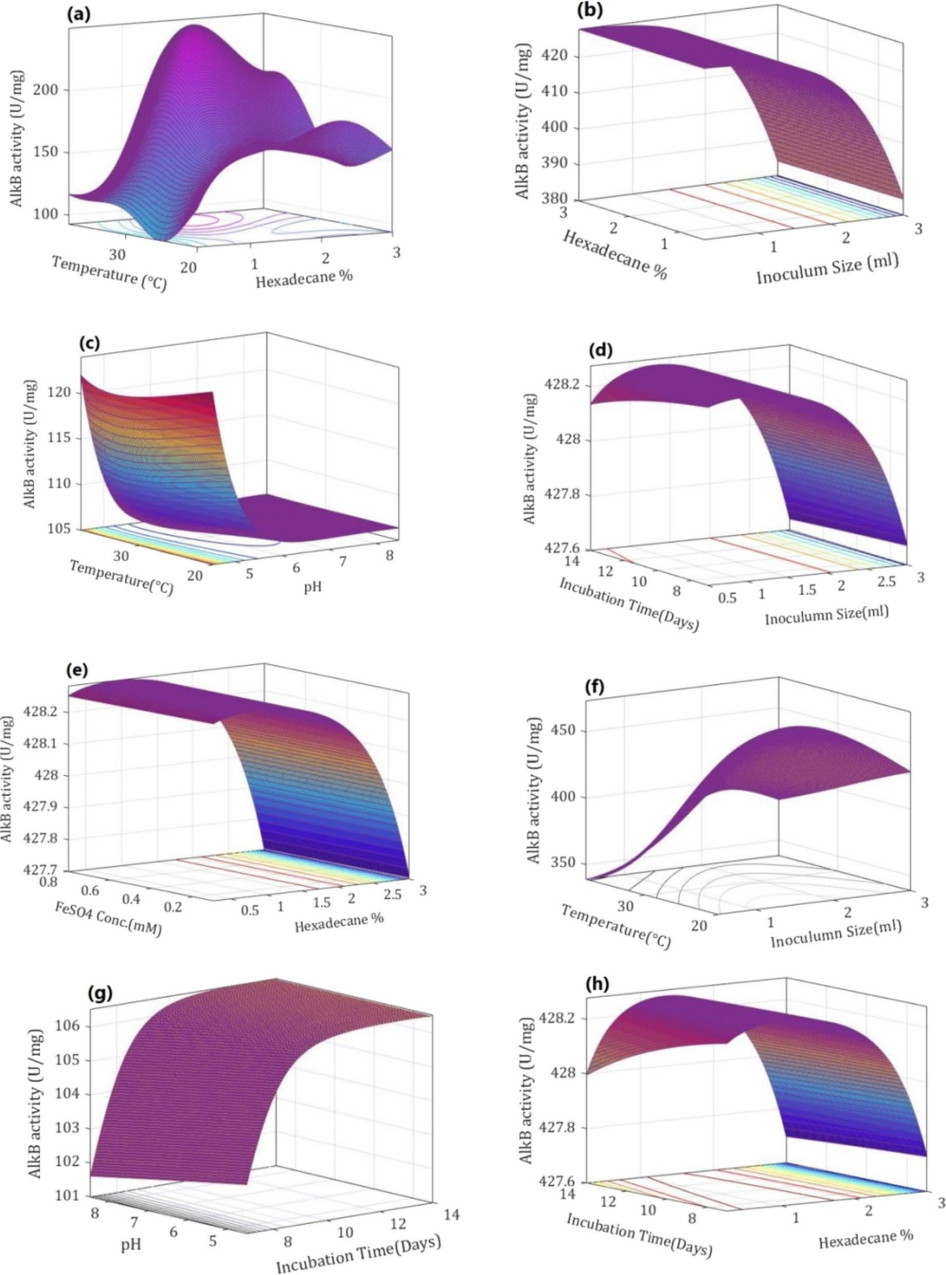
Interactions of fermentation parameters and their effect on AlkB specific activity. **a** Hexadecane *vs* temperature, **b** hexadecane vs inoculum size, **c** pH vs temperature, **d** inoculum size *vs* incubation time, **e** hexadecane vs FeSO_4_ concentration, **f** inoculum size vs temperature, **g** Incubation time vs pH, **h** hexadecane vs incubation time (In above plots, only two variable factors have been indicated on x and y-axis and remaining factors were maintained at optimum levels achieved from 2nd run of GA optimization, which are as follows: hexadecane: 1.556%, Temperature: 27.40 °C, pH: 7.38, Incubation time: 11.56 days, Inoculum size: 1.33 ml per 100 ml media (v/v), FeSO_4_ conc.: 0.63 mM)

Based on the results obtained after FFNN-GA optimization, surface contour plots were generated to examine the impact of one variable on another using fitness function with MATLAB R2020a. The maximum AlkB specific activity observed with the combined effect of two variables at the optimum environment is shown in Fig. 6. The variation in AlkB specific activity could be seen with the variation of hexadecane from 0.25 to 3% and incubation temperature from 20 to 38 °C (Fig. 6a). Maximum AlkB specific activity was observed with 1.5% of hexadecane as carbon source and incubation temperature of 38 °C. Further increase in hexadecane concentration beyond 1.5% led to a decrease in activity, which indicated that hexadecane concentration and incubation temperature together have a higher impact over AlkB production. Figure 6b indicated variation in AlkB specific activity with variation in inoculum size (1–3 ml) and variation in supplementation of hexadecane (0.5–3%) in the medium. The maximum AlkB specific activity was observed up to 1 ml of inoculum size and beyond this, there was a sharp decline in its specific activity. However, hexadecane concentration from 1 to 3% didn’t influence AlkB specific activity much. This indicated that inoculum size played the role of key regulator for AlkB specific activity when varied in combination with hexadecane concentration. The pattern of the contour plot between pH and incubation temperature indicated that temperature in the range of 20–35 °C did not influence AlkB specific activity significantly, whereas pH above 5 has shown a negative impact on it (Fig. 6c). In Fig. 6d combined impact of incubation period and inoculum size on AlkB specific activity is shown, which indicates that less than 8 days of incubation and more than 1.5 ml of inoculum size have the least impact on AlkB specific activity. From Fig. 6e, it can be observed that AlkB specific activity remained constant with 0.1–0.8 mM FeSO_4_ concentration and decreased with hexadecane concentration of more than 1.5%. Interaction of inoculum size (1–3 ml) and incubation temperature (20 °C to 38 °C) showed maximum activity near 30 °C temperature and 2.5 ml of inoculum size (Fig. 6f) and reflected that both parameters were collectively regulating AlkB specific activity. An incubation time (7–11.5 days) with pH (4.5–8.5) together had a positive impact on activity, whereas, an incubation time of more than 12 days didn’t have any impact irrespective of the pH value (Fig. 6g). From Fig. 6h it could be concluded that AlkB specific activity was maximum with 1.5% of hexadecane with 8–12 days of incubation, however, it sharply declined beyond 1.5% of hexadecane. The contour surface plots (Fig. 6) show that each combination/individual fermentation parameter has an effective contribution to the overall AlkB specific activity.

## Discussion

Considering promising industrial applications of AlkB, process parameters were optimized for the production of AlkB from *Penicillium chrysogenum* SNP5. It has shown good growth on hexadecane, and its uptake as a carbon source was confirmed by AlkB specific activity of 100 U/mg and the presence of fatty acids in ferment. Banu et al. (2010) and Kadri et al. (2018) have also reported similar results. Submerged fermentation was chosen because it provides larger surface area and high oxygen availability which assisted proper growth of *Penicillium chrysogenum* SNP5 and facilitated the uptake of hexadecane as a carbon source. With submerged fermentation, separation of cell biomass and extraction of membrane-bound AlkB is easier than solid-state fermentation (Flores-Flores et al. 2011). It is well established that yield of enzymes varies with critical process parameters and microbial strains because the growth and metabolism of microbes are dependent on the various physicochemical environment, nutritional factors and combinatorial impact of various process parameters (Vishwanatha et al. 2010; Saxena and Singh 2011; Narra et al. 2012). Six input variables were considered to study their individual and combined effect on AlkB specific activity through OVAT. Hexadecane was used as a carbon source in the media, as AlkB had shown high specificity for it, hence could act as an inducer for AlkB production. The pH of the media becomes crucial in case of submerged fermentation and AlkB is quite susceptible to pH as well. As AlkB is integral protein (membrane bound), hence its yield is biomass dependant and growth associated, therefore, incubation temperature and period would play critical role in achieving optimum yield. The number of viable cells in the inoculum ensures rapid proliferation and biomass synthesis which results in increased production of enzymes, hence, inoculums size was selected as an input parameter. FeSO_4_ was selected as one of the input parameters because AlkB is a nonheme iron-containing enzyme whose catalytic property is strongly dependent upon iron. Further, data obtained from OVAT were used for FFNN-GA to achieve optimum yield with the limited experimental data.

FFNN-GA optimization showed very promising results in terms of AlkB specific activity. The results obtained from FFNN like correlation charts (Fig. 1), performance plot (Fig. 3) and error histogram (Fig. 4) indicated that training of neural network was done very accurately with network topology of ‘6-12-1’ (Fig. 2). These results are close to the published results (Das et al. 2015; Negi et al. 2020; Suryawanshi et al. 2020). A high degree of accuracy of optimization is attributed to the selection of appropriate training algorithms (i.e., trainlm), several hidden layers and the size of ‘one-variable-at-a-time’ data for the training of the network.

The GA optimization utilizes the FFNN outputs to provide the global optimum solution for non-linear problems. Results obtained after GA optimization (Table 4) clearly indicated that there was significant increase in AlkB specific activity when it was cross-validated with experimental data, which reveals that the used fitness function generated the best fitness values for optimum AlkB specific activity. This could be possible due to significant weights and biases value obtained after network training and selection of appropriate values of GA parameters (i.e., population size, crossover probability, mutation probability and the number of generations, etc.). Similar results have been published by Badhwar et al. (2020) and Prakasham et al. (2011).

The 77.4% significant improvement from FFNN-GA optimization reveals that GA has efficiently used its reproduction function, crossover function and mutation functions to generate the good strings, new populations whereas iteration process might have able to find out the best global optimal solutions. The GA might have identified slight changes in inoculum size, incubation time, pH of the media and metal ion concentration as key regulators and generated the several combinations of these critical parameters to provide the highest yield. Specially, FeSO_4_ has higher impact on enhanced AlkB yield due to its dependency on iron for catalytic activity.

An interactive contour plot generated with the help of GA outputs (Fig. 6) indicated that the best optimum output could be achieved by generating an infinite number of combinations of two input variables keeping other variables at their optimized level. A similar study has also been reported by Salim et al. (2019). Enhanced AlkB specific activity with an increase in hexadecane concentration and incubation temperature (Fig. 6a) suggested that uptake of hexadecane was easier at higher temperatures which induced the higher production of AlkB. On the other hand, the effects of hexadecane and inoculum size on AlkB specific activity (Fig. 6b) suggested that up to 1 ml of inoculum size was sufficient for the utilization of 1–3% of hexadecane, which might be due to a higher percentage of cell viability in a spore suspension. Figure 6c indicated high sensitivity of AlkB towards variation in pH-temperature combo, hence, a slight change in pH from 7 reduced AlkB activity, which might be due to a change in the ionic state of the active site of AlkB. Figure 6d indicates that AlkB production started after 8 days of incubation. This might be due to the availability of simple carbon source (glucose) in the media which supported its initial growth and only after the consumption of glucose present in the media, hexadecane utilization might have started, which resulted in the production of AlkB. An increase in hexadecane concentration decreased AlkB activity (Fig. 6e), which could be due to its toxicity and hydrophobicity. Combination of inoculum size and temperature (Fig. 6f) had shown less impact on the AlkB specific activity beyond inoculum size of 1.5, whereas; incubation time and pH (Fig. 6g) together influenced AlkB specific activity much more. Figure 6h suggested that higher hexadecane concentration reduced the AlkB activity due to excess substrate accumulation of substrate toxicity.

An overall observation from contour plots indicates that specific activity of AlkB was influenced by hexadecane concentration in combination with some other parameters in the following orders: metal ion concentration > incubation time > inoculum size > incubation temperature (Fig. 6). The wide variation in enzyme yield shown in Table 2 (i.e. minimum 39.44 U/mg for 56th set and maximum 198.94 U/mg) emphasizes the significance of the machine learning-based optimization approach for the cost-effective production of membrane-bound enzymes. A similar data (i.e., minimum 71.33 U/ml, maximum 218.28 U/ml), has been reported by Sathish and Prakasham (2010). Specific activity of AlkB was improved by 77.4% (i.e. from 198.94 to 351.32 U/mg) when FFNN output enzyme production data was further optimized using GA. Sathish and Prakasham (2010) also reported a 47% improvement in the yield of glutaminase after ANN output enzyme production data was further optimized using GA. An overall 3.5-fold increase in AlkB specific activity (from 100 U/mg under preliminary un-optimized conditions to 351.32 U/mg after FFNN-GA optimization) has been achieved using FFNN-GA hybrid method in this study. Subba Rao et al. (2008) had also reported more than 2.5-fold improvement in alkaline protease yield using FFNN–GA hybrid methodology. From the statistical observation, the R-  Value of 0.987 of FFNN training exhibited a better correlation between predicted and experimental data with ‘6-12-1’ FFNN topology. The overall smallest values of mean absolute percentage error (MAPE) and root mean square error (RMSE) of 0.053 and 6.801, respectively suggested that chosen network had good approximation and generalization aspects for the optimization of AlkB yield.

From the overall findings in this study, it can be concluded that OVAT strategy alone is not capable to find out optimal conditions for enhancing AlkB yield due to the requirement of a large number of experiments and lack of determination of interactions among various factors. FFNN-GA coupled optimization approach significantly enhanced the AlkB specific activity. The FFNN model ‘6-12-1’ showed the best prediction accuracy after training with the Levenberg–Marquardt (trainlm) algorithm. These findings signify the utility of the FFNN-GA approach for the enhanced production of Alkane hydroxylase from *Penicillium chrysogenum* SNP5 and optimization of other bioprocesses in the enzyme industry.

## Data Availability

The datasets generated during and/or analyzed during the current study are available from the corresponding author on reasonable request.

## Abbreviations

AlkB: Alkane hydroxylase
FFNN: Feed Forward Neural Network
GA: Genetic algorithm
SmF: Submerged fermentation
MAPE: Mean absolute percentage error
RMSE: Root mean square error
MSE: Mean square error
PMSF: Phenylmethylsulfonyl fluoride
OVAT: One variable at a time
TBH: Tetrahydrobiopterin
LDAO: Lauryldimethylamine oxide
NADH: Nicotinamide adenine dinucleotide (NAD) + hydrogen (H)
PDA: Potato dextrose agar
CMC: Critical micelle concentration
